# Improving Tumor Retention of Effector Cells in Adoptive Cell Transfer Therapies by Magnetic Targeting

**DOI:** 10.3390/pharmaceutics12090812

**Published:** 2020-08-27

**Authors:** Laura Sanz-Ortega, José Manuel Rojas, Domingo F. Barber

**Affiliations:** 1 Center for Hematology and Regenerative Medicine (HERM), Department of Medicine, Karolinska Institute, 14183 Stockholm, Sweden; laura.sanz@ki.se; 2 Animal Health Research Centre (CISA)-INIA, Instituto Nacional de Investigación y Tecnología Agraria y Alimentaria, 28130 Madrid, Spain; rojas.jose@inia.es; 3 Department of Immunology and Oncology, and NanoBiomedicine Initiative, Centro Nacional de Biotecnología (CNB)-CSIC, 28049 Madrid, Spain

**Keywords:** magnetic targeting, cancer immunotherapy, adoptive cell transfer therapy, chimeric antigen receptor (CAR) T-cells

## Abstract

Adoptive cell transfer therapy is a promising anti-tumor immunotherapy in which effector immune cells are transferred to patients to treat tumors. However, one of its main limitations is the inefficient trafficking of inoculated effector cells to the tumor site and the small percentage of effector cells that remain activated when reaching the tumor. Multiple strategies have been attempted to improve the entry of effector cells into the tumor environment, often based on tumor types. It would be, however, interesting to develop a more general approach, to improve and facilitate the migration of specific activated effector lymphoid cells to any tumor type. We and others have recently demonstrated the potential for adoptive cell transfer therapy of the combined use of magnetic nanoparticle-loaded lymphoid effector cells together with the application of an external magnetic field to promote the accumulation and retention of lymphoid cells in specific body locations. The aim of this review is to summarize and highlight the recent findings in the field of magnetic accumulation and retention of effector cells in tumors after adoptive transfer, and to discuss the possibility of using this approach for tumor targeting with chimeric antigen receptor (CAR) T-cells.

## 1. Introduction

For many years, conventional treatments such as surgery, chemotherapy and radiotherapy have been the main options to treat cancer. However, the high relapse rates together with the numerous side effects that these therapies produce highlight the need for developing new supplementary treatments to overcome these limitations and problems. In recent years, cancer immunotherapy, which seeks to fight cancer by enhancing the immune response, has re-emerged as one of the most promising therapies to solve these issues [1]. One of the physiological functions of the immune system is to recognize and eliminate altered cells due to tumor transformation. The emergence of a tumor nonetheless implies that the immune response failed to be adequately triggered and this could be due to several non-excluding causes such as (i) the inability to detect tumor-associated antigens (TAAs), (ii) the recognition of tumor antigens as one’s self, creating a regulatory response rather than an effector one, (iii) a reduced infiltration of the effector cells in the tumor, and iv) the presence of immunomodulatory factors that generate an immunosuppressive tumor environment [2]. Antitumor immunotherapy seeks to enhance the response of components of the immune system so that they can overcome the tumor escape mechanisms and establish an effective antitumor response [3,4]. Broadly speaking, cancer immunotherapy strategies can be classified as active or passive [5,6]. Active immunotherapy aims at stimulating the immune system to attack tumor cells through vaccination, non-specific immunomodulation or through the activation of antigen-specific receptors, while passive immunotherapy consists of the administration of agents such as monoclonal antibodies or lymphocytes that would increase the existing antitumor response [7]. This review will focus on adoptive cell therapy (ACT) and how magnetic targeting can improve this passive strategy.

## 2. Adoptive Cell Therapy (ACT) and Its Limitations

ACT involves the isolation of immune cells from a patient or donor, which can be modified and expanded ex vivo under optimal conditions to then be reinfused back to the patient [8,9,10]. This approach is based on the premise that a large population of cells with antitumor activity will migrate to the tumor and mediate its destruction. The first clinical trials using ACT were pioneered by Rosenberg et al., who used lymphokine-activated killer cells (LAK cells), which consisted of interleukin (IL)-2 activated autologous peripheral blood mononuclear cells, along with high doses of IL-2, for the treatment of tumors such as melanoma or colorectal cancer [11]. The clinical benefits in these studies were limited, however, since the high doses of systemic IL-2 induced some severe toxicity [12]. Since then, different cell types, mainly based on T or natural killer (NK) cells, have been investigated for use in ACT.

Although ACT has achieved high regression rates in some types of cancer, there is still a lack of response in other cases [13]. This can be due to numerous factors that affect the transferred cells including their proliferation potential [14,15], their persistence [16], their differentiation state and phenotype [17,18], as well as their ability to migrate and infiltrate the tumor [19,20,21]. Another important limiting factor for ACT success is often related to the failure to isolate and expand in sufficient numbers tumor-specific T cells that mediate tumor destruction [9,22]. Both the time and the cost required to produce these cells are a great challenge [23]. Tumor cell immunogenicity also plays a crucial role in cancer immunotherapy. Response rates to immunotherapeutic approaches (such as programmed-death ligand receptor 1 (PD-1) pathway blockade) increase in tumors with high mutational rates [24,25], indicating that tumor cells carrying a greater amount of mutations are more readily detectable and thus targetable by immunotherapy. Consequently, ACT is more likely to be effective in tumors with high mutation burdens.

It is well known that the trafficking of an adequate number of effector cells to the tumor region is a critical step in the development of an antitumor immune response. This process is dynamic and highly regulated, and the optimal antitumor responses in several cancers positively correlate with an increase in the infiltration of antitumor effector cells [26,27,28,29]. However, it often appears in ACT that, both in humans [30,31] and in mice [32,33], only a small percentage of the transferred cells manage to migrate to the tumor and infiltrate it. These cells also travel indiscriminately to multiple organs [34], and this could have pathological consequences. Therefore, one of the biggest challenges in ACT is to overcome the barriers that restrict the access of effector cells to the tumor niche and design new strategies to increase the traffic of these cells to the tumor microenvironment.

**Figure 1:**
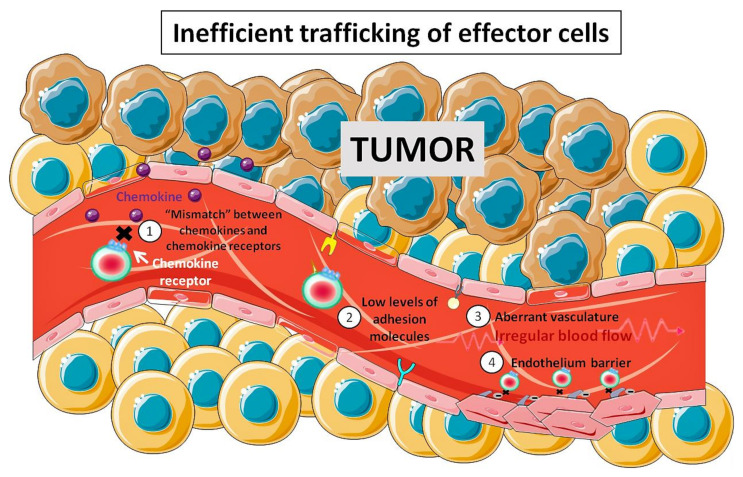
Main barriers that restrict the efficient traffic of effector cells towards tumors. The tumor microenvironment is a hostile environment for antitumor effector cell infiltration. This is achieved through multiple mechanisms such as: (**1**) mismatch between the chemokine receptors and chemokine pool in the tumor microenvironment; (**2**) decrease in the expression of adhesion molecule on the tumor endothelium; (**3**) aberrant vasculature that leads to inefficient traffic of immune cells; (**4**) tumor endothelium acting as a barrier.

Antitumor effector cells are recruited from the circulation to the tumor site by a sequential process involving endothelial adhesion, rolling, chemotaxis and finally, extravasation [35,36]. The reasons for the low trafficking of effector cells to the tumor are still a topic of active research. It is known that multiple mechanisms can prevent the infiltration of effector cells in tumors such as: (i) the mismatch between the chemokine receptors and the chemokine pool in the tumor environment [37,38,39,40], (ii) the decrease in the expression of adhesion molecules, (iii) the aberrant tumor vasculature that can promote irregular blood flow and therefore inefficient traffic of immune cells [41], and (iv) the possible role of the tumor endothelium as a barrier [42]. In summary, tumors are able to create multiple cellular and molecular barriers that restrict the efficient entry of effector cells within the intratumoral regions (Figure 1).

The mismatch between the chemokine receptors expressed by the effector cells and the chemokines secreted by tumors can be responsible for suboptimal traffic of immune cells in the tumor [35]. Numerous chemokines have been described to regulate the migration of T and NK cells towards the tumor. C-X-C Motif Chemokine Receptor 3 (CXCR3) is one of the main receptors that is activated in tumor infiltrating lymphocytes (TILs) in melanoma [38], colorectal cancer [40] and breast cancer [37]. Efficient traffic of cytotoxic T lymphocytes (CTLs) to metastatic sites in melanoma patients correlates with the expression of C-X-C Motif Chemokine Ligand 9 (CXCL9) and CXCL10 which are CXCR3 ligands. This chemokine receptor has been found deregulated in effector T cells [38]. Additionally, not all tumors express enough CXCR3 ligands [37,38], which may result in inefficient recruitment of effector and memory CD8^+^ T cells. CXCR6 is another important chemokine receptor overexpressed in activated T cells. Its absence caused a decrease in T cell infiltration in breast tumors, which prevented tumor regression in a breast cancer model [39]. One option to overcome ineffective homing is to genetically modify the transferred cells to improve their ability to effectively migrate to the tumor. Kershaw et al. demonstrated that the introduction of the CXCR2 chemokine receptor in T cells allowed for their effective migration to tumor cells in vitro [43]. Since then, multiple studies aiming at increasing the traffic of T and NK cells to the tumor through modifications of other chemokine receptors have been published. Di Stasi et al. showed that C-C Motif Chemokine Receptor 4 (CCR4) expression in CD30-directed chimeric antigen receptor (CAR) T cells improved migration towards CD30^+^ Hodgkin lymphoma, which secreted the CCR4 ligand CCL17 (C-C Motif Chemokine Ligand 17). In addition, CCR4^+^ CD30-directed CAR-T cell transfer resulted in superior antitumor activity in a xenotransplant model due to increased infiltration of T cells in the tumor [44].

Another obstacle for T and NK cell tumor infiltration is the presence of an aberrant vasculature in the tumor, which can promote an irregular blood flow leading to inefficient cell trafficking in the tumor microenvironment [41]. In addition, tumor endothelium has been described as a barrier that prevents infiltration of CTLs [42]. To overcome these issues, several strategies such as the use of antibodies against vascular endothelium growth factor (VEGF) and its receptors [41,45,46], irradiation [47], or cytokine targeting (e.g., tumor necrosis factor-α (TNFα)) with tumor vasculature NGR (Asn-Gly-Arg) and RGR (Arg-Gly-Arg) homing peptides [41,48], have focused on the normalization of blood vessels in the tumor to facilitate immune cell infiltration. All of these strategies have been shown to increase tumor infiltration. CAR technology has also been used to target endothelial cell components such as the αV/β3 integrin [49] or the VEGF-2 receptor overexpressed in the tumor vasculature [50] to remove the aberrant vasculature and increase the traffic of effector cells towards the tumor, which in turn could mediate tumor regression.

The efficient traffic of T and NK cells to the tumor environment is critical for developing successful antitumor immunotherapy. Although new strategies that increase antitumor immune cell traffic to the tumor are being investigated, they often rely on specific mechanisms that differ between tumor types. The design of more general approaches, which can be applied to a wide variety of tumors, would be of great interest.

## 3. Nanobiomedicine

Nanotechnology is a multidisciplinary area based on the design and use of materials and systems on the nanometric scale. Biomedicine is one of the areas in which nanotechnology has had a major impact, giving rise to nanobiomedicine. Nanobiomedicine uses nanotechnological systems such as nanoparticles, nanocells, nanoemulsions, etc., for different biomedical applications such as prediction, prevention, early diagnosis and personalized therapy, among others [51]. Nanotechnological solutions can present several advantages over conventional delivery routes: treatments can be controlled and directed more specifically, stability can be prolonged, and degradation delayed. This in turn implies that higher concentrations of the active ingredient in the desired place can be achieved, thus increasing the effectiveness of the treatment and decreasing systemic toxicity.

### 3.1. Nanoparticles as the Base of Localized Delivery System

As previously mentioned, a main limitation in most treatments that use drugs or biomolecules is their low in vivo efficacy. As a result, high concentrations of bioactive agents are used to obtain the desired effect, thus causing multiple adverse effects due to off-target interactions, which can sometimes lead to high toxicity. ACT has the advantage over conventional therapies of presenting low systemic toxicity as it employs tumor specific effector cells. ACT application is, however, limited by two main issues: (i) the difficulty to obtain and expand a high enough number of specific effector cells, and (ii) the dissemination of transferred cells throughout the body. Nanotechnology could represent a two-fold solution to solve these types of problems by reducing the need for cell expansion and by concentrating the effects of ACT. Nanoparticles (NPs) are one of the most commonly used nanosystems in biomedicine to improve therapeutic efficacy. NP-based systems have been mainly used to specifically release drugs or biomolecules. They present two main advantages: (i) their small size allows them to reach certain areas inaccessible to other delivery systems and (ii) they can be directed towards a desired area by active or passive strategies, thus reducing the amount of bioactive agent necessary to achieve therapeutic effects in a given location and consequently minimizing the dose and the systemic toxicity.

These characteristics of nanotechnological solutions are critical when attempting to improve the action of a drug in a specific area, as occurring in cancer or in organ-specific autoimmune disease. Conventional therapies tend to disperse throughout the body with only part of the bioactive agent reaching the site of action. NPs can be directed to the site of action through active or passive strategies [52]. Passive targeting strategies rely on the NP size which usually accumulates passively in inflamed regions or tumor masses due to an increase in permeability in those areas, a phenomenon known as enhanced permeability and retention (EPR) effect [53]. The therapeutic potential of this effect has been illustrated in several preclinical and clinical trials that demonstrated that the binding of antitumor drugs to NPs could increase their effectiveness compared to the administration of the drug alone [54]. Active targeting strategies seek to actively direct the NPs towards the region of interest. Several strategies have been developed for active targeting such as associating the NPs to specific ligands that direct them to a specific tissue type [55]. Another very promising strategy is the use of magnetic NPs (MNPs) that can be precisely located in the desired area by using an external magnetic field (EMF) [56].

### 3.2. Magnetic Nanoparticles

The idea of MNPs as transport agents that could be targeted and concentrated in a specific region by using an EMF was first proposed by Freeman et al. in 1960 [52,57,58], and it is based on the competition between the forces exerted on the MNPs by the blood flow and the EMF applied. When the latter predominates, the MNPs can be retained in the desired area. To be used in biomedicine, it is important that MNPs present several properties that make them biocompatible and prevent their toxicity. In addition, their design properties should be tailored to their final application [57]. In the case of MNPs, these characteristics are defined by the magnetic material of the core as well as by their coating. MNP can be categorized depending on the metallic core (e.g., iron, cobalt, nickel, etc.) and the coating employed for their biological application.

The magnetic material must provide essential inherent characteristics, such as the superparamagnetic behavior, to fit the NP’s intended use. In superparamagnetic materials, fluctuations in the direction of magnetization affect the entire particle, as these have a single magnetic domain due to their small size, which makes their magnetic behavior reversible when applying a magnetic field. In larger particles, for instance for iron metallic cores >20 nm, the magnetic behavior is not reversible. In biomedicine, the concept of superparamagnetism is especially relevant, since in the absence of an EMF, the material does not have a residual magnetization, thus avoiding the attraction and agglomeration between particles, which could cause problems such as embolization in the bloodstream. Another important consideration is that the magnetic core needs to be biodegradable or easily excreted. NPs with iron cores can be processed by cells by using the biochemical pathways of iron metabolism [59,60,61]. Finally, the magnetic core must provide high magnetization, so that the movement of the NPs can be controlled with an EMF and can be immobilized in the desired region. The present review will focus on iron oxide NPs, as these have been most actively researched due to their biocompatibility.

MNP coating will provide NPs with additional characteristics tailored for their use [62]. Coating MNPs with biocompatible compounds prevents their possible toxicity, reduces their immunogenicity and increases their residence time in blood. Besides, the coating can provide the MNPs with the ability to bind certain compounds when a targeting moiety is used. Furthermore, when the MNP coating presents a surface charge at physiological pH, it allows stabilization and prevents aggregation due to the repulsion forces between charges of the same sign.

MNP coating can be achieved through adsorptive or covalent linking to the metallic core (reviewed in [63]). A wide range of polymer formulations has been described to confer stabilizing properties to the MNPs in biological fluids and can allow grafting of supplemental functionalizing biological molecules if required [64]. This endows MNPs with great versatility since they can be chemically manipulated to become vehicles capable of transporting magnetically their cargo to the site of interest.

### 3.3. MNP for ACT

As previously mentioned, ACT is a very promising therapeutic approach for cancer treatment. NP-based strategies have been used to modify T cell activity to promote antitumor activity [65,66]. NP-modified T cells have even been employed as a chemotherapy carrier for disseminated tumors [67]. Although these strategies proved effective, they can be technically challenging. They also do not address one of the main limitations of ACT, i.e., the reduced migration and the inefficient infiltration of effector T and NK cells in the tumor microenvironment. Therefore, there is a clear need for strategies that allow one to increase the traffic of the transferred cells to the tumor, thus allowing an effective antitumor action and complete tumor eradication [68]. MNP application in biomedicine can improve the effectiveness of a treatment and decrease its toxicity, so combining MNP use with ACT is a very attractive idea. Attaching MNPs to effector lymphoid cells used for ACT to guide them to the tumor site with the application of an EMF is therefore a promising concept. Moreover, increasing the EMF application time or repeating EMF application could also improve the magnetic targeting [56]. Several studies using this type of strategy have been carried out, but mainly with the objective of accumulating stem cells, macrophage or mesenchymal cells as well as dendritic cells (DCs) in regenerative therapies and autoimmune disorders [69,70,71,72,73,74]. Its use with lymphoid cells, such as T or NK cells for cancer or autoimmunity treatments, has been very limited [75].

Unlike DCs and macrophages, effector cells such as T and NK cells are characterized by their high motility and for being continuously in circulation. The magnetic targeting of these effector cells could improve their migration to the tumor and promote their accumulation and infiltration in it. This strategy, if implemented correctly, could solve one of the main problems of ACT.

The application of an EMF could lead to an increase in the adhesion time of these cells in the region of interest and, therefore, in their infiltration within the tumor. Only a few studies have addressed the influence of MNPs on the migration and functionality of immune system cells [76]. As promising as this strategy appears, it would only serve as long as the key functional aspects of effector cells are not affected by the presence of MNPs and the application of an EMF.

We and others have recently demonstrated the feasibility of this approach and analyzed the effects of the combined use of magnetic nanoparticles and the application of an external magnetic field, to promote the accumulation and retention of lymphoid cells [77,78,79,80,81]. The following sections of this review will highlight some of the recent findings on the effects of MNPs on lymphoid cells, their specific targeting through the application of external magnetic fields and the possibility to use this platform to solve some of the limitations such as the low efficiency in lymphocyte trafficking, that some current antitumor immunotherapies present.

## 4. The Interaction between MNPs and Lymphoid Cells

In spite of the attractiveness of MNP-based targeting of effector cells in ACT, some basic considerations must be fulfilled for these applications to be successful: (i) MNP must not show toxicity towards the lymphoid cells; (ii) lymphoid cells must be able to interact with the nanomaterial; (iii) finally, the nanomaterial must be easy to produce in large amounts so that the numerous immune cells required for ACT can be loaded. The use of the co-precipitation synthesis method, which is simple and easily reproducible and scalable, offers a simple solution to this last point [82].

There are several studies reporting that the toxicity of MNPs depends on their coating, the cell type they are interacting with, the MNP concentration, and the exposure time [83,84,85]. In general, MNPs do not cause notable toxicity in different immune cell types, with high cell viability percentages often observed [86]. MNP toxicity usually occurs mainly when high doses and prolonged exposure times are used [87,88] or in the presence of certain coatings [89]. Besides, there are several studies demonstrating the low toxicity of MNPs in both human and murine T and NK cell lines and in primary T and NK cells [75,78,80,89,90,91,92,93,94,95,96,97,98,99].

We have, however, observed an increase in the mitochondrial metabolic capacity of these cells after association with MNPs [78]. This could indicate some non-specific activation by MNPs, since an increase in mitochondrial mass and activity is usually related to the activation of T and NK cells [100,101,102], sometimes due to the presence of reactive oxygen species (ROS) [103], a feature often induced by the presence of MNPs in cellular systems [104,105]. In contrast, this was not observed in expanded murine NK and activated CD8^+^ T cells. Since these cells are already in an active state, it is probably more difficult to induce or detect significant changes in this aspect.

**Figure 2:**
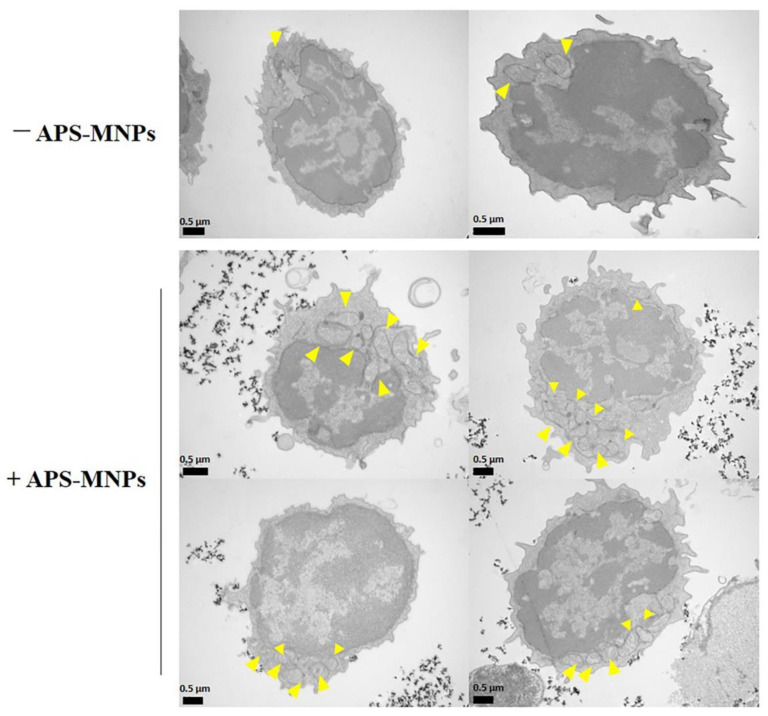
MNP treatment increases the presence of mitochondria in naïve T cells. Representative images obtained by transmission electronic microscopy from murine naïve T cells with MNPs. Arrowheads indicate the mitochondria within the cells. TEM methodology is described in [78].

Further analyses of the metabolic state of primary T cells revealed that they had increased mitochondrial respiration and glycolysis in the presence of MNPs [78]. These features could be associated with an activated state in T cells, since aerobic glycolysis increases during the transition from naïve to effector T cells and the proliferation that takes place after the genetic rearrangement of the T cell receptor (TCR) [106]. In addition, TEM imaging of primary T cells showed an increased presence of mitochondria after treatment with MNPs (Figure 2). Since these cells are barely metabolically active and present a low number of mitochondria in the early stages, these data indicate that MNPs could promote some metabolic activity in resting lymphoid cells. In line with this observation, Mühlberger et al. observed a slight decrease in the percentage of naïve T cells after MNP treatment, both in resting cells and in polyclonally-stimulated cells, hinting that MNP loading may facilitate T cell activation [81].

It has been recently reported that activation of pattern recognition receptors (PRR), which include Toll-like receptor (TLRs) and play a role in the initiation of innate immune responses, can regulate TCR signals and T cell functions [107]. We have previously shown that some MNP coatings such as polyethylenimine (PEI) can trigger macrophage activation through TLR4 signaling [108]. The responsiveness to PRR signaling thus indicates that T cell activation could be altered by MNP treatment. This is a new research area that will require further investigation.

**Figure 3:**
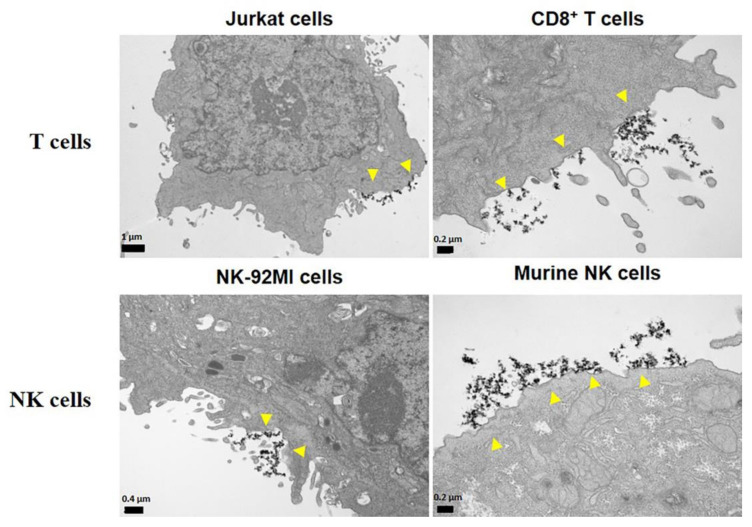
Subcellular localization of the APS-MNPs in different lymphoid cell models. Representative images obtained by transmission electronic microscopy. Arrowheads indicate the MNPs associated with the cell membrane.

Studies of the subcellular localization of MNPs showed that, unlike macrophages, mesenchymal or tumor cells, which are able to internalize different types of MNPs [56,70,108,109,110], lymphoid cells are apparently unable to do so (Figure 3) [78,79,111,112]. It is widely described that the uptake of NPs depends on the cell type [113,114]. In the case of lymphoid cells, the MNPs often remained on the cell surface, in close contact with the plasma membrane. Lymphoid cells typically have a low phagocytic capacity [115] which would limit the entry of MNPs into the cytoplasm. Indeed, special MNP designs, such as functionalization with RGD (Arg-Gly-Asp) or tat peptides, are often required to improve internalization by these non-phagocytic cells [88,94,116,117,118]. Other strategies such as transfection or targeting with anti-CD3 antibodies have been attempted to improve MNP labeling of T cells [112,119]. Although effective for lymphoid cell loading, MNP functionalization complicates synthesis and cell handling which would introduce increased variability in MNP-lymphoid cell preparation.

Indeed, we and others have described that lymphoid cell loading with minimally functionalized MNPs could be sufficient to achieve magnetic retention [78,79,81]. Our microscopy analysis and iron uptake measurements showed that positively charged MNPs (3-aminopropyl-triethoxysilane (APS)-MNPs) were most associated with all types of lymphoid cells [78]. It is well described that positively charged NPs interact to a greater extent with cell membranes, possibly due to electrostatic interactions [114,120,121,122,123,124,125,126]. Muhlberger et al. used negatively charged MNPs and found sufficient association with T cells to elicit retention by a magnetic field, indicating that MNP interactions with lymphoid cells are not solely dependent on charge [80,81]. The activation status of the lymphoid cells is also likely to affect its capacity to interact with MNPs. We reported that freshly isolated primary T cells associated with MNPs to a lesser extent than activated T cells [78]. This observation corroborated another study that showed that after in vitro stimulation lymphoid cells were able to incorporate more NPs [88].

Overall, most studies evaluating MNP interactions with lymphoid cells indicate that MNPs will be minimally taken up by lymphoid cells. As such, the design of MNPs for loading on lymphoid cells should rely on optimization of the MNP surface charge to increase interaction with the cell plasma membrane (e.g., produce positively charged MNPs that interact with negatively charged membrane lipids). An improved understanding of the mechanics of interaction between plasma membrane and MNPs will surely lead to the design of better MNPs for loading on lymphoid cells in the future.

## 5. In Vitro Functionality in MNP-Loaded Lymphoid Cells

A sine qua non condition for MNP-based targeting strategies in ACT to be effective is that the effector lymphoid cells retain their function when loaded with MNPs. Several studies consistently indicate that the expression of surface markers is not significantly affected by MNP presence in a wide variety of cells including lymphoid cells [71,72,78,80,96,127,128,129]. It thus appears that minimally functionalized MNP (i.e., with a simple chemical coating that allows stable colloidal suspension) do not significantly alter the activity of lymphoid cells.

There are, however, surprisingly few studies systematically assessing the effect of MNPs on the multifaceted functionality of effector lymphoid cells. It has been described in in vitro studies for both human and murine T cells that their proliferation to specific antigens, their cytotoxic capacity against certain cell targets and their cytokine production were not affected by MNP treatment [95,96,130,131]. Our studies which looked at multiple parameters to assess T cell functionality after MNP treatment confirmed these findings [78]. Studies also showed that MNP-treated effector T cells maintain their cytotoxic and antitumor activity in vivo and can infiltrate the tumor without apparent problem [94,132]. However, defects in the cytolytic capacity and cytokine production of these cells have also been described, but only in the presence of very high doses of MNPs [88]. Another study showed that dimercaptosuccinic acid (DMSA)-coated MNPs altered cytokine induction and affected the Kv1.3 channel activity in Jurkat cells [133]. This indicates that, similar to other cell types, MNP treatment of lymphoid cells has the potential to interfere with redox activity. Overall, most studies indicate that MNPs association with effector T cells does not significantly alter T cell functions.

Only few studies have evaluated MNP effects on NK cells, and most have only addressed this question using human NK cell lines such as NK-92 or KHYG-1. These studies show that NK cell lines preserve their cytolytic capacity in vitro and are able to migrate and infiltrate the tumor similarly to cells not associated with MNPs in vivo [75,97,98,99,134,135]. Our group recently reported the effect of MNP treatment in several functional aspects of NK cell biology in two NK cell models: a human NK cell line and primary murine NK cells [79]. NK cell recognition of its target is mediated by a range of receptors capable of detecting their ligand expressed in transformed/stressed/infected cells, which leads to cytotoxic responses and/or cytokine production by this effector cell [136]. For NK cell effector functions to take place, the NK cell needs to first conjugate to its target so that an immunological synapse forms to mediate the NK cell cytotoxic responses. In the case of cytokine responses, target recognition should lead to the activation of signaling pathways that result in cytokine production.

**Figure 4:**
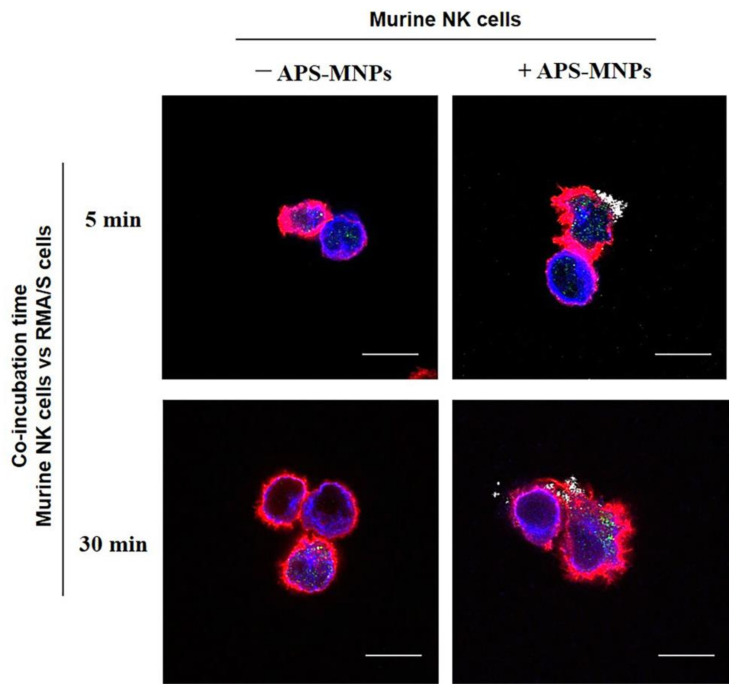
Conjugation of murine NK cells with the RMA/S cell target in presence of MNPs. NK cells were treated with MNPs (+ APS-MNPs) or left untreated (− APS-MNPs) and incubated with RMA/S target cells for different lengths of time. Representative images of their conjugation at different incubation times, acquired by confocal microscopy (actin (red), microtubules (blue), perforin (green), MNPs (gray)). Scale: 10 μm. Dark-field confocal imaging is described in [78] and conjugation technique in [79].

MNP presence on the cell membrane of NK or T cells did not affect the effector cell conjugation capacity to target cells in two-color flow cytometry [77,79]. Since MNP can affect cytoskeleton remodeling [137,138,139,140], we also assessed by confocal microscopy the formation and polarization of the microtubule organizing center (MTOC) in the synapse between primary NK cell and target cells (Figure 4). No defects in the NK cell MTOC were observed, indicating that MNP-loaded NK cells can conjugate normally to their target cells and form an immunological synapse that would allow effector functions to be triggered.

NK cell-mediated killing of target cells is usually mediated by a process called degranulation that refers to the release of lytic granules containing enzymes that mediate target cell killing. Measuring this process can be used as a surrogate for the cytotoxic capacity of NK cells (and cytotoxic T lymphocytes) [141]. We found that MNP treatment increased in a dose-dependent manner the spontaneous degranulation of NK cells [79], but not of CD8^+^ T cells [77]. The degranulation of MNP-loaded NK cells after stimulation also increased compared to unloaded cells, but this was quantitatively similar to the increase in baseline degranulation levels. We also observed increased degranulation in MNP-loaded CD8^+^ T cells when compared to unloaded cells in presence of antigen. These findings indicate that degranulation could be enhanced by the presence of MNP on the surface of cytotoxic cells. In spite of this, an increase in cytotoxicity towards target cells was not detected as both MNP-treated NK cells and T cells could lyse target cells similarly to untreated cells [77,79]. Other reports also indicate that NK cell MNP treatment does not alter the cytotoxic ability of these cells [75,88,96,98,132,134].

Another important functional aspect of NK cell biology is their capacity to produce cytokines that modulate immunity [142]. Among those, interferon (IFN)-γ is one of the most important pro-inflammatory cytokines produced by this type of cell [143]. We and others have reported that MNP treatment did not affect IFN-γ production in human or murine NK cells, as well as CD8^+^ T cells, in response to various stimuli [77,79,88,95,131]. NK cells have been reported to produce other cytokines and chemokines that modulate immunity, such as IL-5, IL-10 or CCL5 for instance [144,145,146,147]. Assessing the production of these cytokines in the presence of MNPs would be useful in the future to better understand the capacity that MNPs could have in altering alternative NK cell responses to stimuli.

NK cells are continuously circulating and are recruited from the circulation to the tumor by a five-step sequential process that involves: (1) cell rolling on the endothelium followed by (2) a chemokine-activation step that results in (3) activation and full adhesion to the endothelium. The adhered cells then (4) migrate laterally to find a site where they can penetrate the endothelium to finally (5) cross the endothelial barrier [35,36]. These characteristics are integral to NK cell functionality, and it is therefore important to assess MNP effects on these mechanisms. We reported that NK cells treated with APS-coated MNPs could still adhere and transmigrate through an endothelial monolayer [79]. In fact, the presence of MNPs in the cell membrane occasionally increased adhesion to endothelial cells. This could be due to the MNP exposition on the membrane of NK cells that could ease their interaction with the endothelium due to the MNP charge. These results are in line with several other studies which show that both T and NK cells associated with MNPs could migrate and infiltrate the tumor tissue in vivo as effectively as untreated cells [94,96,97,98,99,132,134]. Overall, it appears that MNP treatment of T and NK cell produce minimal alterations in their function. MNP presence on the cell surface could slightly increase basal degranulation of cytotoxic cells, but other functions such as cytotoxicity, cytokine production or transmigration through the endothelium seem to remain intact. Although it remains critical to evaluate the activity of each MNP preparation on lymphoid cells, most studies indicate that the effects of minimally functionalized MNPs could be negligible on these cell populations, thus opening the gate to MNP use as magnetic targeting agents for lymphoid cells.

## 6. In Vitro and In Vivo Magnetic Retention of MNP-Loaded Immune Cells

### 6.1. In Vitro Magnetic Retention

**Figure 5:**
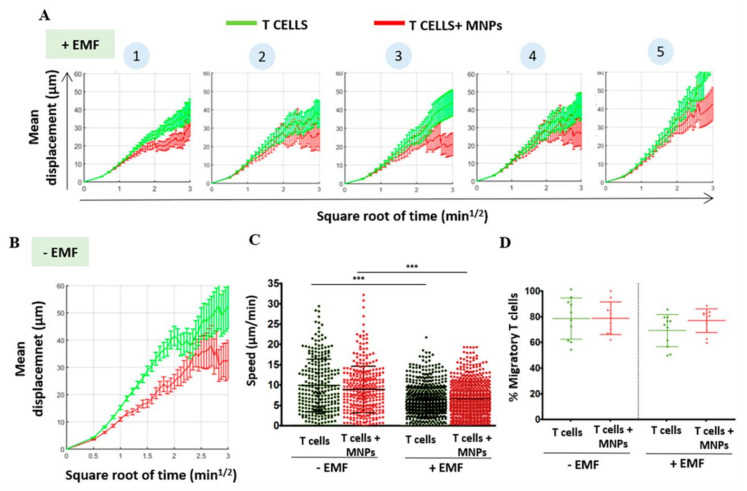
Analysis of the migratory capacity of murine T cells after association with MNPs and in presence of an EMF, in in vitro “under agarose” migration assays. Coverslips were coated with recombinant murine Intercellular Adhesion Molecule 1 (ICAM-1) and CCL21 and overlaid with agarose. Primary murine T cells were labelled with CellTracker orange dye (Invitrogen), injected under the agarose layer with a micropipette and allowed to polarize for 30 min prior to the EMF application. Five positions (1 to 5) were selected with the microscope at increasing distance of the applied magnet (position 1 being the closest to the magnet and position 5 being the furthest from the magnet), and T cell movement was tracked over time. Mean displacement versus time graphs of T cells in the “under agarose” assay (**A**) at increasing distances from an EMF and (**B**) in the absence of an EMF. On one hand, these results indicate that MNP treatment by itself produces alterations in the migratory capacity of murine T cells, which show a reduction in the displacement along the time compared to untreated cells. On the other hand, EMF impairs migration in both MNP-treated and untreated murine T cells in a distance-dependent manner. Quantification of (**C**) cell speed and (**D**) percentage of cells that migrate in all conditions after analyzing the movies using Imaris software. The results shown (mean ± SD) are representative of two independent experiments. Student’s t-test, *** *p* < 0.001. These results show that both MNP treatment and EMF application reduce the velocity of murine T cells. However, no differences in the number of cells that was able to migrate were found in the different conditions.

The chemotactic response is critical and essential for the trafficking of lymphoid cells through the different tissues and regions where they are required. We reported that the association of lymphoid cells with MNPs slightly decreased in some cases the response by these cells to a chemotactic gradient. This effect could be corrected by the application of an EMF in the same direction as the chemotactic gradient [78]. Similar results have been described in DCs [69,72], indicating that the loading of immune cells with MNPs could affect chemotaxis. In vitro “under agarose” migration assays, which allow the study of polarized T cell behavior within a confined system, showed that the presence of MNPs and EMF affected cell migration (Figure 5). MNP-loaded T cell mobility was reduced when compared to unloaded counterparts even in the absence of EMF (Figure 5B). EMF application further reduced MNP-loaded cell migration in these studies, and this effect was dependent on the distance at which the EMF was applied. EMF applied at closer proximity to the migration system (i.e., equivalent to increasing the magnetic field) limited MNP-loaded T cell displacement further than when placed at distal location (Figure 5). Curiously, a similar effect was also detected in unloaded T cells, albeit to a lower extent. Several factors could explain the reduction in migration produced by MNP loading. On the one hand, there are several studies showing that the presence of MNPs in cells can affect the rearrangement of the actin cytoskeleton and microtubules, thus limiting their motility [137,138,139,140]. Although in these experiments, the MNPs were associated with the plasma membrane, and not in the cytoplasm, so the decrease in migration is unlikely due to a direct effect of MNPs on the actin machinery. On the other hand, it has been observed that the presence of magnetic components such as MNPs and/or the exposure to moderate static magnetic fields can influence biological systems mainly through alterations of the transmembrane ionic fluxes, such as the calcium ion flux [148,149,150]. It is known that changes in intracellular calcium levels govern rapid cytoskeleton remodeling, which is key in T cell activation [151], as well as T cell migratory response to chemokine gradients or their extravasation, among other processes [152]. This could also explain this “non-specific” retention effect on unloaded cells and will be discussed in a subsequent section of this review.

MNP-based retention of lymphoid cells in the organism requires balancing the strength of the EMF with the flow forces that are applied to these circulating cells. In vivo MNP-based retention will therefore only be effective if lymphoid cells can be retained in a specific location in spite of the presence of these flow forces. We showed that a minimum amount of MNPs needs to be associated with the cells for them to be retained by an EMF. Magnetic retention increased with the amount of MNPs associated with the cells as well as with the force of the magnetic gradient applied [78]. Others also found that similar amounts of MNP were necessary to load lymphoid cells to magnetically retain them [80,81]. Indeed, additional studies confirm that three parameters are critical for magnetic targeting: (1) amount of MNP associated to cells; (2) force of the magnetic gradient; (3) length in time of EMF application [73,153].

### 6.2. In Vivo Retention of MNP-Loaded Cells in Lymphoid Tissues

Magnetic in vivo tissue targeting with MNP-loaded cells has been reported, but they usually involved other cell types, mostly macrophages or mesenchymal cells [69,70,71,72,73,74]. Recent reports extend to lymphoid cells the application of magnetic targeting to specifically promote cell accumulation—an area of research often overlooked until now [75,77,78]. We reported that EMF application favored the retention of MNP-loaded T cells in the lymph node (LN), confirming that magnetic targeting is achievable in vivo with MNP-loaded lymphoid cells [78]. Interestingly, the mere presence of MNPs on the cell surface increases the retention of these cells in secondary lymphoid organs. However, T cells associated with MNPs did not show a preferential location or distribution towards a specific lymphoid organ in the absence of EMF. This preferential accumulation of MNP-associated T cells in lymphoid tissues appears to be the result of decreased in vivo cell mobility.

Multiple factors could explain the increased retention of MNP-loaded cells in secondary lymphoid organs. It has been previously described that cells associated with MNPs present a greater tendency to aggregate [154]. It appears that MNPs could act as a link that facilitates cell-to-cell interactions, probably due to their electrostatic charges. This phenomenon could contribute to the reduced speed of MNP-loaded cells in LN. Indeed, in vivo multiphotonic microscopy studies showed that T cells associated with MNPs have a reduced speed in the LN [78]. This could contribute to the increased retention of these MNP-associated cells in lymphoid tissues, even in the absence of an EMF, as the reduction in speed could prolong their interaction with tissue vasculature and facilitate retention.

EMF application could also affect cell speed. It has been demonstrated that moderate static magnetic fields can influence biological systems primarily through alterations at the level of transmembrane ionic fluxes and of the fluidity of the phospholipids that constitute cell membranes [148,149,150]. Microscopy studies of calcium flux showed that maximum and mean responses were reduced in murine T cells after MNP treatment, which could indicate that the presence of magnetic components in cell membranes could affect calcium signaling [78]. This fact is important since it has been described that the magnitude and duration of calcium fluxes influence the basal velocity of T cells in LNs [151]. A reduced amplitude in intracellular calcium peaks related to arrest, or more limited T cell movement, in order to favor their interaction with the stroma and with other leukocytes [155,156]. Therefore, since the magnitude and duration of intracellular calcium levels control T cell responses, EMF application could affect essential biological aspects of these cells such as activation [151] or migration in response to chemotactic gradients [157]. These are important consideration that will need addressing for using MNP as directional agents in ACT.

It has also been reported that the biological effects caused by a magnetic field depend more on the magnitude of the magnetic gradient than on the strength of the field [158]. Indeed, when two EMF were used around an LN (thus increasing the magnetic gradient) we observed a further reduction in T cell speed in the LN [78]. This phenomenon also occurred with unloaded T cells, indicating that strong magnetic gradient could alter T cell circulation speed in lymphoid organs. Analyses of the cell trajectories in these experiments indicated that the presence of MNPs did not significantly alter the path of the lymphocytes within the LN when an EMF was applied, although a study in greater depth, at longer times, and with a higher EMF could be necessary to detect changes in the trajectory due to the tissue complexity of the LN.

**Figure 6:**
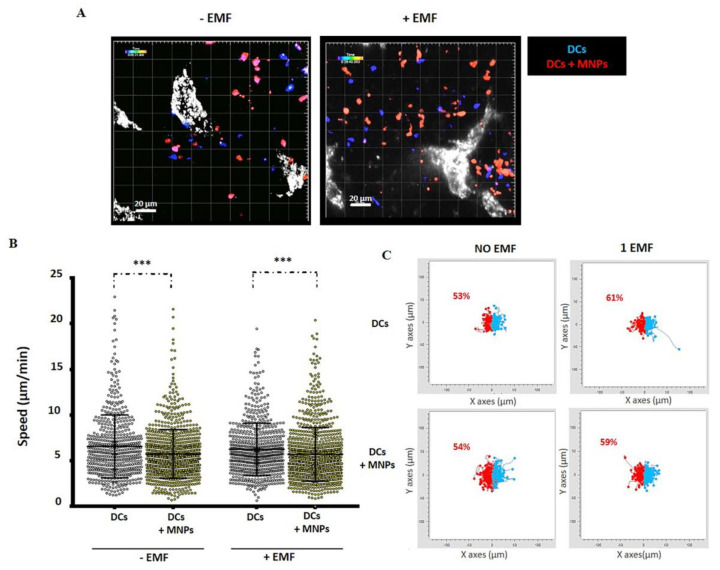
In vivo behavior of MNP-loaded or unloaded murine DCs in the popliteal LN in the presence or absence of an EMF. (**A**) Representative captures of multiphoton microscopy movies obtained in presence or absence of EMF (blue: MNP-free DCs, red: MNP-associated DCs, gray: high endothelial venules (HEVs)). (**B**) Quantification of cell speed after movie analysis using Imaris software. The results shown (mean ± SD) are representative of two–three independent experiments. Student’s t-test, *** *p* < 0.001. (**C**) Paths followed by MNP-treated or untreated DCs inside the LN during the multiphoton microscopy assays in the absence or presence of EMF placed on the left (overall DC displacement to the left in red; to the right in blue), analyzed with the software provided by Ibidi (Chemotaxis and Migration tool). The methodology and ethic committee permits for these experiments are detailed in [78].

In a pilot study, we loaded dendritic cells (DCs) with MNPs, as these cells have a greater capacity for MNP internalization than T cells [69,72,159,160] and thus a greater potential for attraction to an EMF (Figure 6). This also allowed us to determine whether MNP internalization in immune cells promoted the same retention phenomena in secondary lymphoid organs that is described for membrane-loaded T cells. As for T cells, a greater number of DCs loaded with MNPs were found in the popliteal LN. It thus appears that the presence of MNPs can increase the retention or infiltration of immune cells within lymphoid organs independently of the MNP cellular location (Figure 6A). A decrease in cell velocity in LN was also observed for MNP-loaded DCs, but not for unloaded cells (Figure 6B), and this appears to be independent of the presence of EMF in our experimental setting. Several reports have shown that MNP-labeled DCs are retained in LN when adoptively transferred [71,161]. Retention of MNP-loaded DCs in LN could therefore be favored by the reduced speed these cells display when loaded with nanomaterial. Cell trajectory analysis in LN showed a slight increase in DC migration towards the EMF side (Figure 6C). Multiple parameters could explain the reduction in the speed of MNP-loaded DCs in the LN. MNP presence can affect the cytoskeleton reorganization after internalization [137,138,139,140] and EMFs have the potential to alter transmembrane ion fluxes and/or the membrane phospholipids organization among other things [148,149,150]. These factors could alter the activation status and migratory capacity of DCs, and more in-depth studies would help to better understand the in vivo behavior of DCs associated with MNPs and in the presence of an EMF.

Overall, it appears that, independent of the presence of MNPs on the cell surface or intracellularly, loading of immune cells is likely to increase the retention time in lymphoid organs. It is worth noting that delivery routes of DC-based vaccines that target LNs are associated with improved antitumor effects [162,163]. This characteristic of MNP-loaded immune cells could therefore be exploited for therapeutic ends. Similarly, T cells associated with MNPs can be magnetically retained in the LNs and this could potentially be used to modulate T cell activity in these lymphoid organs to control pathology. For example, in a model of autoimmune ovarian disease, the accumulation of regulatory T cells in the ovary-draining LN counteracted the pathogenic immune response and inhibited the development of the disease [164]. Conversely, the retention of DCs in the LN could also be useful in cancer vaccination to trigger specific tumor antigen responses. Several studies have explored strategies to accumulate DCs in the LNs using MNPs [69,71] some of which improve immunotherapy [71]. The duration of the interaction between DCs and T cells in the LN modulates the specificity and strength of the T cell response [165,166,167,168,169], so the decrease in the speed of MNP-loaded T cells and DCs that we report in LNs, could increase their interaction time and consequently trigger more powerful T cell responses with greater affinity. This MNP-based strategy could also be applied to other regions of interest, such as a tumor, with the aim of increasing the residency time of effector cells in the targeted tissue and thereby enhancing antitumor immune responses.

## 7. In Vivo Magnetic Tumor Targeting of Lymphoid Cells: Not So Straightforward

Most studies concur that MNP loading does not cause alterations or defects in lymphoid cell functions. Thus, using this loading strategy to direct cells to a site of interest with an EMF appears as an attractive and feasible approach to improve ACT effectiveness. Surprisingly few reports have, however, employed this strategy to improve ACT in a tumor context. Jang et al. demonstrated using the NK cell line, NK92-MI, labeled with fluorescent MNPs, that application of an EMF could enrich adoptively transferred cells in the vicinity of the tumor although they did not study their effects on tumor growth [75]. We have recently contributed to this field by assessing the magnetic retention of antigen-specific CD8^+^ T cells in vivo in a tumor model and determining whether tumor magnetic targeting improved ACT-based treatment. We used a murine syngeneic tumor model, where the implanted tumor cells express ovalbumin (OVA) as a surrogate tumor antigen which can be specifically recognized by MHC class I-restricted, OVA-specific CD8^+^ T cells (OT-I cells). In this model, tumor growth is reduced by OT-I CD8^+^ T cell infiltration [94,170,171]. This allowed us to examine whether the application of an EMF over the tumor could favor the retention of MNP-loaded OT-I CD8^+^ T cells in that region. Surprisingly, we were unable to increase the accumulation of antigen-specific CD8^+^ T cells at the tumor site in our experimental setting [77]. In fact, EMF application appeared detrimental to therapy with MNP-loaded antigen-specific CD8^+^ T cells. Adoptive transfer of MNP-loaded antigen-specific CD8^+^ T cells did nonetheless achieve the same therapeutic effect as unloaded cells in the absence of an EMF, indicating that MNP-loading did not impair normal migration to the tumor site. Other ACT studies using MRI to visualize OVA-specific CD8^+^T cells loaded with MNPs also found that these cells maintain their baseline tumor homing activity [94,132]. Subsequent tissue analysis revealed a greater infiltration of MNP-loaded antigen-specific CD8^+^ T cells in the tumor-draining LN of EMF exposed mice. Additionally, these cells presented a higher activation status. These results indicate that EMF exposure can favor the retention of the transferred antigen-specific CD8^+^ T cells in the tumor-draining LN instead of the tumor. This is an important finding for the field, as it illustrates that magnetic forces will compete with a multitude of biological factors that can interfere with the delivery of lymphoid cells to their intended destination. Indeed, because of the proximity between the LN and the tumor, the exposure to an EMF could have favored the retention of these MNP-loaded cells in the LN, a more suitable niche for T cells than the tumor microenvironment. The observation that the antigen-specific CD8^+^ T cells in this LN present a more activated phenotype confirms that their residency time in this tissue was increased [77], as longer T cell interactions in secondary lymphoid tissues usually correlated with increased activation. The application of one or more smaller magnets such as “magnetic needles”, which provide a more focused magnetic field in a limited area [172], could be used to improve specific infiltration into the tumor and not in the LN. Another approach to increase magnetic targeting was recently proposed by Pai et al., which showed that dynamically programmable magnetic fields improved MNP-labeled CAR-T cells’ spatial targeting [111]. These data highlight the complexity of translating in vitro observation to in vivo systems, in which a plethora of physical and biological processes will compete with magnetic forces.

Our report indicates that adoptively transferred MNP-loaded antigen-specific T cells could become trapped in LNs proximal to the tumor mass and this could be problematic for effector cell delivery at the tumor site. It is therefore of great interest to try to understand why this T cell retention occurred in the draining LN rather than in a tumor that presented a greater magnetic field. As previously discussed, the reduction in speed of MNP-loaded T cells in LNs could prolong their interaction with the lymphoid tissue vasculature and facilitate their retention. In addition, the tendency of the cells that carry MNPs to aggregate [154] could contribute to the inability to leave the LN.

Besides the physical factors related to the presence of MNPs on these antigen-specific CD8^+^ T cells, biological aspects of the LN structure could also favor the retention of these cells. Sphingosine-1-Phosphate Receptor 1 (S1PR1) expression on T cells is a master regulator of effector T cell egress from the LN [173,174,175,176,177]. It has been described that after TCR stimulation, the expression of S1PR1 decreases [174], then, a mechanism mediated by the overexpression of the activation marker CD69 complexes with S1PR1, causing its internalization and degradation [174,178,179]. This probably increases the interaction time of activated T cells in the LN, which promotes their differentiation into effector cells, onto which S1PR1 is again upregulated to permit their egress from the LN. Alteration in S1PR1 expression in T cells could therefore limit the capacity of adoptively transferred cells to exit the LN they infiltrated. Furthermore, it has also been described that antigen-specific regulatory T cells exert their immunosuppressive action in the LNs by promoting, among other things, a decrease in the expression of S1P1R in effector T cells, thereby trapping these cells in the LN [180]. Evaluating the expression of S1PR1 and of other chemokine receptors, such as CCR7 [181], that control T cell migration in adoptively transferred cells would therefore be useful to optimize the migratory profile of transferred MNP-loaded T cells, so that their retention in the tumor is not only promoted by the EMF but also by biological factors such as chemokines.

Specific antigen recognition by the TCR decreases T cell migration and favors T cell contacts with the antigen presenting cells (APCs) [182]. T cell activation results in a change in cell polarity that decreases its speed and increases its adhesion capacity, mainly through Lymphocyte function-associated antigen 1 (LFA-1) and other integrin expression [183]. This inhibition in activated T cell migration is dependent on Rac1 and promotes retention at the site of inflammation and infection [184]. T cell migration within the LN is dependent on the formation of various structures (lamellipodia and uropod) that allow cell displacement, by reorganizing cytoskeleton components [185,186]. However, some of these elements can only be mobilized to promote one cellular function, such as the microtubule organizing center (MTOC) that has a different orientation depending on whether the cell is migrating or interacting with APCs [182]. Thus, an imbalance between the formation and relocation of certain cytoskeleton structures can also produce a decrease in migration.

Enhanced interactions with resident LN cells (e.g., macrophages) or even with the LN extracellular matrix due to MNP presence could also play a role in the apparent retention of loaded cells in LNs. For instance, subcapsular and spinal macrophages within the LN are involved in the entrapment of gammadelta (γδ) T cells in LNs for long periods of time [187]. In addition, interactions between lymphocytes and the extracellular matrix are usually transient when the lymphocyte has a high migration capacity, since the formation of focal adhesions inversely correlates with the speed of migration [188]. The reduced intranodal speed of transferred T cells when associated to MNPs could therefore be due in part to their interaction with certain cell types, such as macrophages or reticular fibroblasts, or even with the extracellular matrix [185], thus hampering their correct transit through the LN [174,189,190]. Multiple biological factors could therefore contribute to the apparent retention of MNP-loaded T cells in the LN. Retention in the LN is an important issue in ACT, and a better understanding of the mechanism governing the trafficking of adoptively transferred cells will surely improve the efficiency of these therapies, independently of the use of MNPs and EMF to promote accumulation at the site of interest. Modulation of lymphocyte trafficking could also find some applications in the treatment of autoimmune diseases [191]. Indeed, the drug, FTY720, designed to block the egress of autoreactive T cells from LNs to prevent their invasion to inflamed regions, has been approved by the FDA for the treatment of multiple sclerosis [192], illustrating that modulation of T cell migration has therapeutic potential.

## 8. Conclusions and Future Perspectives in the Use of CAR-T Cells

This review highlights the recent findings on the effects of MNPs on lymphoid cells, their specific targeting through the application of an EMF and the possibility to use this strategy to target transferred cells to the tumor and thus solve one of the main limitations of ACT in cancer. Most studies show that MNP treatment has minimal effects on lymphoid cell functionality in vitro and in vivo [75,77,78,79,94,96,97,98,99,130,131,132,134].

Key questions, however, remain unanswered concerning basic biological aspects of the interaction of lymphoid cells with MNPs. We have discussed at length the decrease in speed of lymphoid cells when associated to MNP in the LN, which is likely due to a combination of biological and physical factors that increase the interaction time of MNP-loaded lymphoid cells with the resident cells of the secondary lymphoid tissues. Another yet unexplored area would be to evaluate the adjuvancy and metabolic effects that MNPs could have on effector T or NK cells. MNPs have been reported to interact with PRR in immune cells [108,193,194] and we found that mitochondrial metabolism increased in T cells treated with MNPs. Designing MNPs that would favor T cell activation as well as promoting their magnetic targeting could represent an attractive perspective for the future of ACT therapy in cancer. Indeed, the use of engineered MNP as a platform for T cell activation and enrichment has proved successful [195].

Although lymphoid cell retention in the LNs due to MNP loading and EMF application can be problematic for antitumor therapy, this phenomenon could prove useful in certain autoimmune pathologies to prevent effector cell migration towards the affected tissue. Accumulation of tumor-specific CD8^+^ T cells in the tumor-draining LN may have an upside as it could protect against metastasis spreading via the LNs. ACT could also be combined with other strategies, such as those aimed at the normalization of the tumor vasculature, to improve treatment efficiency. These therapies could further facilitate the infiltration of the transferred cells within the tumor by improving the structure as well as the function of the tumor vessels.

Since it is difficult to obtain and/or generate/expand patients’ tumor-infiltrating lymphocytes and tumor-specific T cells in sufficient numbers, the use of effector T or NK cells modified with engineered TCRs or chimeric antigen receptors (CAR) has become a powerful approach within ACT therapies. Engineered TCR therapy involves the introduction in T cells of cloned TCRs with high affinity for a TAA, thus redirecting T cell activity towards cancer cells carrying the TAA. For example, there are specific cloned TCRs against the Melanoma Antigen Recognized by T cells 1 (MART-1) that can be introduced into human lymphocytes to specifically treat patients with positive tumors for this antigen [196]. Unfortunately, one of the main tumor escape mechanisms to evade the activity of T cells is the decrease in major histocompatibility complex (MHC) molecule expression [197], thereby limiting antigen presentation and antitumor responses induced by TCR-engineered cells. CARs would solve this immunoevasion problem since T cells are endowed with artificial receptors specific for a surface tumor protein or antigen [198]. There are currently a variety of studies demonstrating CAR-T cell use in antitumor immunotherapy [199,200,201,202]. Specifically, numerous clinical trials using T-cells with CARs directed against CD19 in B-cell lymphomas have shown remarkable and lasting responses [203], and the FDA has approved two of these therapies in 2017 for clinical use. Regarding the use of NK cells in ACT therapies, modifications of NK cells, such as the NK-92 cell line, with CARs in order to overcome some tumor escape mechanisms and to direct NK cell activity more specifically have been developed [204,205]. The use of MNPs together with localized EMFs could improve the targeting of these genetically modified lymphoid cells and enhance antitumor immune response. An example of this type of strategy has been recently published by Nie et al. In this work magnetic nanoclusters loaded with PD-1 antibody and bound to effector T cells were used as a platform to magnetically recruit effector T cells and PD-1 antibody simultaneously to the tumor with MRI guidance [206] resulting in inhibition of tumor growth in a murine model.

It would also be important for the application of this nanotechnology to develop synthesis methods that could be easily scaled-up and standardized as well as performed in a hospital clean room to allow for their implementation in clinical settings. Clinical-grade MNPs could be prepared together with the CAR-T cells in compliance with Good Manufacturing Practices (GMPs) and their combined use could therefore be more easily translated to clinical practice. The microwave method for MNP production represents a promising approach to this issue as it produces highly uniform nanoparticles in particularly short times with better reproducibility than other strategies and without using toxic materials [207].

As exposed in this review, nanotechnological solutions have the capacity to improve ACT therapy. Controlling immune cell traffic using MNP and EMF would have great potential in the treatment of a diversity of diseases. However, much effort remains in this area to fully understand the mechanisms that govern the interaction of MNP with lymphoid effector cells, and to further apply this technology in vivo where magnetic forces will compete with the complex biological regulation of lymphoid cell migration.
